# Fission yeast strains with circular chromosomes require the 9-1-1 checkpoint complex for the viability in response to the anti-cancer drug 5-fluorodeoxyuridine

**DOI:** 10.1371/journal.pone.0187775

**Published:** 2017-11-09

**Authors:** Hossain Mohammad Shamim, Yukako Minami, Daiki Tanaka, Shinobu Ukimori, Johanne M. Murray, Masaru Ueno

**Affiliations:** 1 Department of Molecular Biotechnology, Graduate School of Advanced Sciences of Matter, Hiroshima University, Higashi-Hiroshima, Japan; 2 Genome Damage and Stability Centre, School of Life Sciences, University of Sussex, Brighton, United Kingdom; Cornell University, UNITED STATES

## Abstract

Thymidine kinase converts 5-fluorodeoxyuridine to 5-fluorodeoxyuridine monophosphate, which causes disruption of deoxynucleotide triphosphate ratios. The fission yeast *Schizosaccharomyces pombe* does not express endogenous thymidine kinase but 5-fluorodeoxyuridine inhibits growth when exogenous thymidine kinase is expressed. Unexpectedly, we found that 5-fluorodeoxyuridine causes S phase arrest even without thymidine kinase expression. DNA damage checkpoint proteins such as the 9-1-1 complex were required for viability in the presence of 5-fluorodeoxyuridine. We also found that strains with circular chromosomes, due to loss of *pot1*^*+*^, which have higher levels of replication stress, were more sensitive to loss of the 9-1-1 complex in the presence of 5-fluorodeoxyuridine. Thus, our results suggest that strains carrying circular chromosomes exhibit a greater dependence on DNA damage checkpoints to ensure viability in the presence of 5-fluorodeoxyuridine compared to stains that have linear chromosomes.

## Introduction

**Fig 1:**
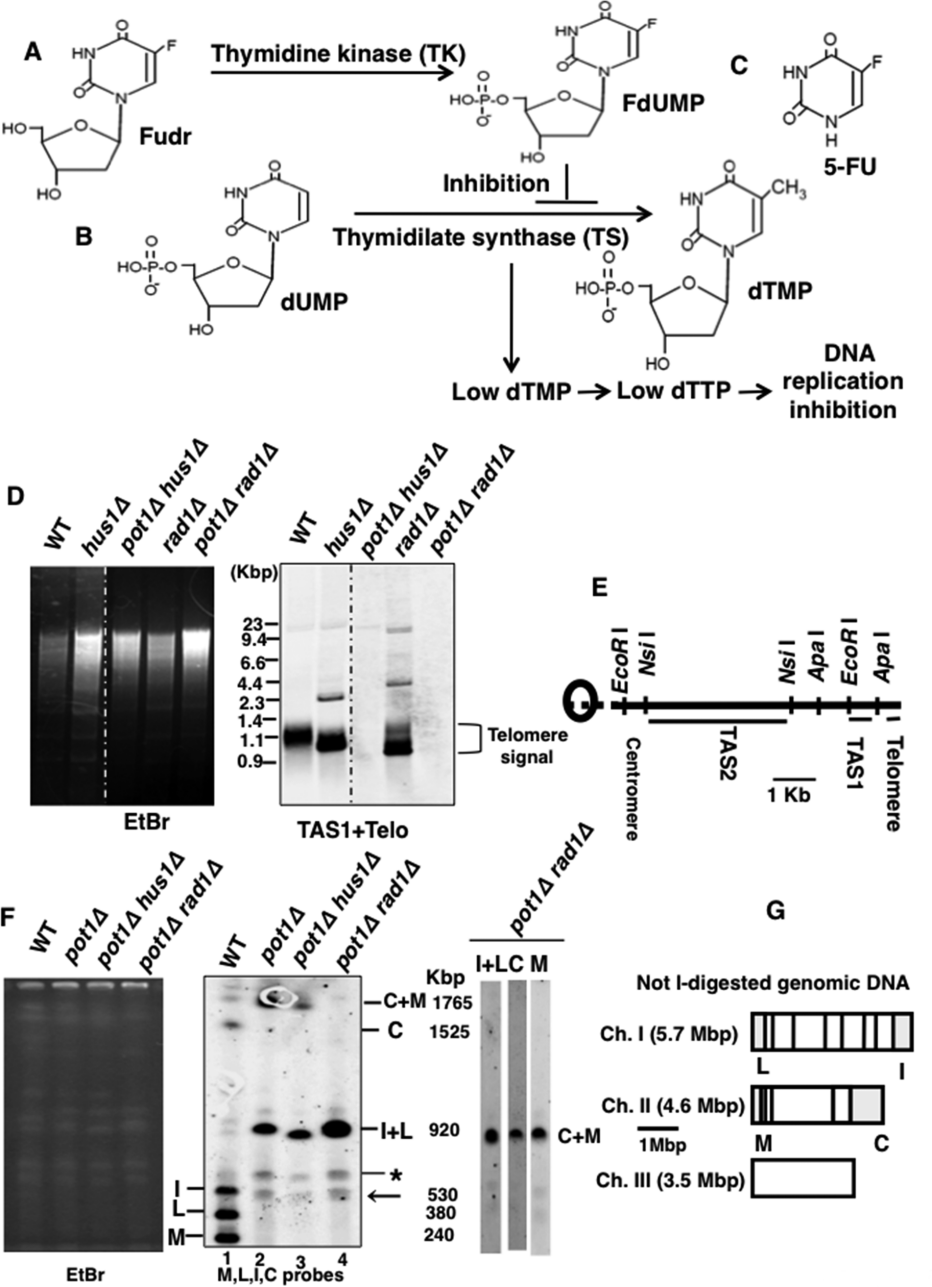
*pot1Δ hus1Δ* and *pot1Δ rad1Δ* cells exhibit telomere loss and circularized chromosomes. **(A)** Fudr conversion to FdUMP by thymidine kinase (TK) [3]. **(B)** In cell, dUMP is converted to dTMP by thymidilate synthase (TS) but FdUMP inhibits the thymidilate synthase resulting in no or very low amounts of dTMP and dTTP production that hamper the DNA replication process [3]. **(C)** Chemical structure of 5-FU. **(D)** The telomeres of wild-type (WT), *hus1****Δ***, *rad1****Δ***, *pot1****Δ*** *hus1****Δ*** and *pot1****Δ*** *rad1****Δ*** cells were analyzed using Southern hybridization at 30°C. Genomic DNA was digested with *Eco*RI and separated by 1.5% agarose gel electrophoresis. A DNA fragment containing telomeric DNA was used as a probe [18]. The Ethidium bromide (EtBr) image shows approximately the same amount of DNA is loaded into the all lanes. Bands with strong telomere signal are denoted ‘Telomere signal’. The weak bands above the telomere signal are either non-specific bands or telomere bands that are not fully digested by *Eco*RI. Sizes of marker are shown. **(E)** Diagram of restriction enzyme sites around the telomere and telomere associated sequences (TAS1 and TAS2) of a chromosome arm cloned in the plasmid pNSU70 [18]. The scale bar corresponds to 1 kb. **(F)** (Left) EtBr stained PFGE agarose gel. (Middle) *Not*I-digested *S*. *pombe* chromosomal DNA from the wild-type (WT), a *pot1****Δ*** isolate, a *pot1****Δ*** *hus1****Δ*** isolate, and a *pot1****Δ*** *rad1****Δ*** isolate were analyzed by PFGE. Probes for the telomeric *Not*I fragments (M, L, I, and C) were used [19]. The asterisk indicates a non-specific band present in all lanes. The arrow indicates a non-specific band present only in lanes 2, 3, and 4. The weak band corresponding to the size similar to C+M signal in lane 1 is a non-specific band. The size of chromosome end fragments digested by *Not*I, M, L, I, C, I+L, and C+M, are shown [19]. (Right) Probes for the telomeric *Not*I fragments (C, M, I+L) were used separately to show that the C+M signal in *pot1****Δ*** *rad1****Δ*** double mutant overlaps with L+I signal. (**G**) *Not*I restriction site map of *S*. *pombe* chromosomes. Chromosomes I, II, and III (Ch. I, Ch. II, and Ch. III) are shown. The scale corresponds to 1Mpb.

DNA replication relies on the availability of deoxyribonucleoside triphosphates and replication fidelity is dependent on their balanced ratios. Deoxyuridine monophosphate (dUMP) is converted to deoxythiamine monophosphate (dTMP) in the presence of thymidylate synthase (TS) [1]. 5-fluorodeoxyuridine (Fudr) is phosphorylated to FdUMP by thymidine kinase (TK) (Fig 1A). FdUMP acts as an inhibitor of TS thereby hampering the synthesis of dTMP and dTTP (Fig 1B). The imbalanced dNTP synthesis consequently negatively impacts on DNA replication and induces DNA damage [2,3]. Accordingly, Fudr and 5-FU are used as a cancer chemotherapy agent to induce double stranded DNA breaks [4] (Fig 1C).

DNA damage activates cell cycle checkpoint signaling pathways, which are crucial for maintaining the cellular integrity by arresting the cell cycle, inducing apoptosis, and repairing DNA. Specifically, in response to DNA damage or replication inhibition (intra S phase checkpoint), unique sensor proteins including the ataxia-telangiectasia mutated-Rad3-related kinase (ATR), ataxia telangiectasia-related-interacting protein complex (ATRIP), and the Rad9-Hus1-Rad1 (9-1-1) complex recognize and bind to the damaged DNA [5] and block cell cycle progression [6,7]. The 9-1-1 complex is a heterotrimeric DNA clamp conserved in human, *Schizosaccharomyces pombe*, and *Saccharomyces cerevisiae*, where the functional analogs are called Rad17, Ddc1 and Mec3 respectively [8]. The 9-1-1 complex structurally resemblance to a sliding clamp, required for replication, proliferating cell nuclear antigen (PCNA) and is loaded onto DNA analogously to PCNA by a specialized clamp loader complex, Rad17-replication factor C (RFC) [9]. Eukaryotic RFC complexes consist of five subunits, which for replication are RFC1-5. For the DNA damage response the large subunit RFC1 is replaced by the cell cycle checkpoint protein Rad17 forming the Rad17-RFC2-5 complex [10]. After DNA damage, single-strand (ss) DNA generated either via resection of the DNA double-strand break or replication-fork stalling, becomes bound by replication protein A (RPA). RPA stimulates Rad17 to bind ssDNA, resulting in the loading of the 9-1-1 complex to this site [11]. The Rad17-RFC2-5 complex then binds to the 3´end of the DNA and uses ATP to open the ring of the 9-1-1 complex so that it can encircle the DNA [8]. TOPBP1 (*S*. *pombe* Rad4/Cut5) bridges between the 9-1-1 complex and the independently loaded ATR-ATRIP complex to promote checkpoint signaling.

DNA integrity is also maintained by telomeres, which comprise DNA-protein complexes located at the ends of eukaryotic chromosomes. Pot1, which is conserved from yeasts to humans, is essential for telomere protection. In the fission yeast *S*. *pombe*, deletion of *pot1*^*+*^ causes immediate telomere loss and chromosome circularization [12]. Circular chromosomes are found in many eukaryotes and circular chromosomes in human have been linked to some genetic disorders and cancers [13]. *S*. *pombe* cells that have circular chromosomes are sensitive to MMS, an alkylating agent that leads to damage in S phase [14]. However, the reasons for this sensitivity to replication stress are not well understood. Here, we investigated the effect of Fudr on fission yeast strains that exhibit defects in DNA damage checkpoints and/or have circular chromosomes. We show that, even though fission yeast does not express endogenous thymidine kinase, the checkpoint-defective *hus1****Δ*** single mutant is sensitive to Fudr. Notably, a *hus1****Δ*** strain with circular chromosomes (*pot1****Δ*** *hus1****Δ*** double mutant), exhibits greater sensitivity to Fudr than each single mutant. Our findings reveal that Fudr causes DNA replication arrest and induces DNA damage and that the 9-1-1 complex is required for viability upon exposure to Fudr and especially in strains with circular chromosomes.

## Materials and methods

### Strain construction and growth media

The strains used in this study are listed in Table 1. The *pot1****Δ*** *hus1****Δ*** double mutant, which carries a plasmid encoding pot1 (*pPC27-pot1*^*+*^*-HA*, *ura4*^*+*^, SH001), was created by deleting *hus1*^*+*^ in strain YI002 by replacement with the *hus1*::*LEU2* DNA cassette amplified from strain SW794. The *pot1****Δ*** *rad1****Δ*** double mutant, which carries a plasmid encoding *pot1*^*+*^ (SH003), was constructed by deleting *rad1*^*+*^ in strain YI002 by replacement with the *rad1*::*LEU2* cassette amplified from strain KT108. YEA plates containing 2 mg/ml 5-Fluoroorotic acid (FOA) at 25°C were used to select for loss of *ura4*^*+*^ and removal of the *pot1*^*+*^ plasmid to obtain *pot1****Δ*** *hus1****Δ*** and *pot1****Δ*** *rad1****Δ*** double mutants (SH002 and SH004). The *pot1****Δ*** *rad9****Δ*** double mutant, which carries the pot1 plasmid (*pPC27-Leu-pot1*^*+*^*-HA*, *tk*, SH009), was generated by deleting *rad9*^*+*^ from SH007 by integration of the *rad9*::*ura4* DNA cassette amplified from SH008. This was plated on YEA plates containing 100 μM Fudr and incubated at 36°C to remove the pot1 plasmid to obtain the *pot1****Δ*** *rad9****Δ*** double mutant (SH010). To detect RPA in *hus1****Δ*** (FY18394) and *pot1****Δ*** *hus1****Δ*** (SH002) cells the large subunit, encoded by *rad11*, was tagged with monomeric red fluorescent protein (mRFP) at the C terminus, the pFA6a-mRFPnatMX6-rad11 plasmid was linearized with *Nsp*V and transformed into (FY18394) and (SH002) to create SH005 and SH006 respectively [15].

**Table 1:** *Schizosaccharomyces pombe* strains used in this study.

Stain name	Genotype	Source or reference
YI002	*h*^*-*^ *ade6 leu1-32 ura4-D18 pot1*::*kanMX6 pPC27-pot1*^*+*^*-HA*	[15]
SW794	*h*^*-*^ *ade6 leu1-32 ura4-D18 rqh1*::*kanMX6 hus1*::*LEU2 rad3*::*ura4*^*+*^ *his7*	Shao-Win Wang
SH001	*h*^*-*^ *ade6 leu1-32 ura4-D18 pot1*::*kanMX6 hus1*::*LEU2 pPC27-pot1*^*+*^*-HA*	This study
SH002	*h*^*-*^ *ade6 leu1-32 ura4-D18 pot1*::*kanMX6 hus1*::*LEU2*	This study
KT108	*h*^*+*^ *leu1-32 ura4-D18 ade6-M210 rad1*::*LEU2 tel1*::*ura4*^*+*^	Our lab Stock
SH003	*h*^*-*^ *ade6 leu1-32 ura4-D18 pot1*::*kanMX6 rad1*::*LEU2 pPC27-pot1*^*+*^*-HA*	This study
SH004	*h*^*-*^ *ade6 leu1-32 ura4-D18 pot1*::*kanMX6 rad1*::*LEU2*	This study
1D	*h*^*+*^ *leu1-32 ura4-D18 his2-245 ade6-M216*	T. Toda
KTA037	*h*^*-*^ *leu1-32 ura4-d18 ade6 pot1*::*kanMX6*	[15]
FY18372	*h*^*-*^ *rad1*::*LEU2 leu1-32 ura4-D18*	NBRP
FY18394	*h*^*-*^ *hus1*::*LEU2 leu1-32 ura4-D18*	NBRP
TN004	*h*^*+*^ *rad11-mRFP*::*natMX6*	[15]
KTA038	*h*^*-*^ *leu-32 ura4-D18 ade6 pot1*::*kanMX6 rad11- mRFP*::*natMX6*	[15]
SH005	*h*^*-*^ *hus1*::*LEU2 leu1-32 ura4-D18 rad11-mRFP*::*natMX6*	This study
SH006	*h*^*-*^ *ade6 leu1-32 ura4-D18 pot1*::*kanMX6 hus1*::*LEU2 rad11-mRFP*::*natMX6*	This study
SH007	*h*^*+*^ *pot1*::*kanMX6 leu1-32 ura4-D18 ade6-M210 (pPC27-Leu-pot1*^*+*^ *-HA)*	Our lab stock
SH008	*h*^*-*^ *leu1-32 ura4-D18 ade6 rad9*::*ura4*^*+*^	Our lab stock
SH009	*h*^*+*^ *pot1*::*kanMX6 rad9*::*ura4*^*+*^ *-D18 ade6-M210 (pPC27-Leu-pot1*^*+*^ *-HA)*	This study
SH010	*h*^*+*^*pot1*::*kanMX6 rad9*::*ura4*^*+*^	This study

### Yeast growth and flow cytometry

After overnight culture of *S*. *pombe* cells in YEA media, one sample of cells was immediately stained with propidium iodide (PI) (0 h) and the remaining samples were taken after 1, 2 and 3 h incubation with Fudr (300 μM). Cells were fixed in cold 70% ethanol in 50 mM sodium citrate, and then the samples were treated with 10 μg/ml RNase A and stained using 2.5 mg/ml PI [16]. Samples were sonicated and then analyzed by fluorescence activated cell sorting (FACS) using a Becton Dickinson FACS Calibur.

### Analysis of telomeres

Telomeric sequences were detected by Southern hybridization using an AlkPhos direct kit module (GE Healthcare), according to a previously described procedure [17,18].

### Pulsed-field gel electrophoresis (PFGE)

PFGE was conducted as in [17,19]. For the observation of *Not*I-digested chromosomes, (*Not*I-digested *S*. *pombe* chromosomal DNA) was fractionated on a 1% agarose gel with 0.5% TBE (50 mM Tris-HCl, 5 mM boric acid, and 1 mM EDTA [pH 8.0]) buffer at 14°C utilizing the CHEF Mapper PFGE system at 6 V/cm (200 V) and a pulse time of 60 to 120 s for 24 h. DNA was visualized by staining with ethidium bromide (1 μg/ml) for 30 min.

### Microscopy

Microscope images of living cells, plated on a glass-bottom dish (Iwaki) coated with 5 mg/ml lectin from Bandeiraeasimplicifolia BS-I (Sigma), were acquired using an AxioCamdigital camera (Zeiss) connected to an AxioObserverZ1microscope (Zeiss) with a Plan-Apochromat 63% objective lens (numerical aperture, 1.4). Pictures were analyzed using AxioVision Rel.4.8.2 software (Zeiss).

### Lactose gradient cell cycle analysis

100 ml cultures of *S*. *pombe* cells were grown overnight to mid log phase (5x10^6^cells/ml) in YEA media. Lactose gradients were made by freezing 10 ml aliquots of a 20% lactose solution in a clear 15 ml Falcon tube and thawing for 1 h before use. Cells were harvested at 3000 rpm for 3 min and resuspended in 750 μl water before slowly adding to the top of the gradient using a cut off blue tip. The gradients were then centrifuged at 1000 rpm for 8 min. Small G2 phase cells were collected by taking out about 0.1–0.4 ml from just below the top of smear of cells using a cut off blue tip. Cells were pelleted at 13000 rpm for 30 sec spin in an microtube and resuspended in 500 μl media, and incubated in YEA liquid medium with Fudr 300 μM at 30°C. Cell cycle progression was monitored by sampling every 20 min from 0 to 300 min, staining with diamidino 2- phenylindole (DAPI) and scoring the percentage of septated and mitotic cells under fluorescence.

### Statistical analysis

Data from two independent experiments were subjected to one-way analysis of variance (ANOVA) followed by Duncan’s multiple range tests. Analyses were performed using statistical applications and differences were considered significant at an alpha level of 0.05. The statistical program used was Stat-View^R^ 5.0 (Mind Vision Software, Abaccus, Concepts, Inc. Berkeley, CA, USA).

## Results

### Both *pot1Δ hus1Δ* and *pot1Δ rad1Δ* double mutants completely lose telomeric DNA and exhibit circularized chromosomes

To examine the role of 9-1-1 complex in the maintenance of circular chromosomes, we created a *pot1****Δ*** *hus1****Δ*** double mutant. The *pot1*^*+*^ deletion leads to a complete loss of telomeric DNA, thus survival relies on the circularization of chromosomes [20]. In contrast, a *pot1* null mutant expressing *pot1*^*+*^ from a plasmid has linear chromosomes and behaves like wild-type cells. If the 9-1-1 complex is required for the maintenance of circular chromosomes, a *pot1****Δ*** *hus1****Δ*** double mutant would not be viable. Therefore, we created a *pot1****Δ*** *hus1****Δ*** double mutant with linear chromosomes, due to expression of *pot1*^*+*^ from a plasmid. We then selected for loss of the *pot1*^*+*^ expressing plasmid on YEA containing FOA. *ura4*^*+*^ cells accumulate a toxic intermediate on FOA so only cells that had lost the *pot1*^*+*^ expressing plasmid, which carries the *ura4*^*+*^ gene, can survive. We successfully obtained viable *pot1****Δ*** *hus1****Δ*** double mutants that had lost the *pot1*^*+*^ expressing plasmid. We also obtained the *pot1****Δ*** *rad1****Δ*** double mutant using same strategy. These results demonstrate that loss of 9-1-1 components Hus1 and Rad1 is not lethal with *pot1****Δ***.

The *pot1*^*+*^ disruptant that has circular chromosomes loses telomeric DNA completely [20]. We next performed Southern blotting to confirm the loss of telomere signal in the *pot1****Δ*** *hus1****Δ*** and *pot1****Δ*** *rad1****Δ*** double mutants, indicative of chromosome circularization. We probed for telomere sequences in wild-type, *hus1****Δ*** and *rad1****Δ*** single mutant and *pot1****Δ*** *hus1****Δ*** and *pot1****Δ*** *rad1****Δ*** double mutant strains. Only linear chromosomes show the telomere signal and *hus1****Δ*** and *rad1****Δ*** single mutants showed very strong telomere signals consistent with having linear chromosomes, as previously reported [21]. In contrast, we observed no telomeric signal in both the *pot1****Δ*** *hus1****Δ*** and *pot1****Δ*** *rad1****Δ*** double mutants consistent with complete loss of telomeric DNA (Fig 1D and 1E). These results suggest that the *pot1****Δ*** *hus1****Δ*** and *pot1****Δ*** *rad1****Δ*** double mutants have circular chromosomes.

For the further confirmation of the circular chromosomes we examined the chromosome structure by pulse field gel electrophoresis (PFGE) of chromosome fragments generated by *Not*I digestion. Fragments M, L, I, and C, which map to the ends of chromosomes I and II, were seen in the wild-type cells but not in a *pot1****Δ*** strain with circular chromosomes or in the *pot1****Δ*** *hus1****Δ*** and *pot1****Δ*** *rad1****Δ*** double mutants. Instead, C+M and L+I bands were detected in the *pot1****Δ*** control strain and the *pot1****Δ*** *hus1****Δ*** double mutant (Fig 1F and 1G). Thus, this result indicates that the chromosomes of the *pot1****Δ*** *hus1****Δ*** double mutant were circularized. We also detected the L+I band in the *pot1****Δ*** *rad1****Δ*** double mutant. However, we did not detect any C+M signal for *pot1****Δ*** *rad1****Δ*** double mutant in the expected position. To test the possibility that C+M band overlaps with L+I signal in *pot1****Δ*** *rad1****Δ*** double mutant, we performed probing using C, M and I+L probe separately and observed strong signal for both probes in the same position. This result suggests that the size of C+M signal is same as that of L+I signal (Fig 1F). We suggest that the novel size of the C+M band is due to chromosome rearrangement which occurred during breakage-fusion-bridge cycles after telomere uncapping [22, 23]. Overall, results are consistent with both *pot1****Δ*** *hus1****Δ*** and *pot1****Δ*** *rad1****Δ*** double mutants carrying circular chromosomes.

### *pot1Δ hus1Δ* and *pot1Δ rad1Δ* double mutants are sensitive to Fudr and Fudr treatment leads to replication arrest

**Fig 2:**
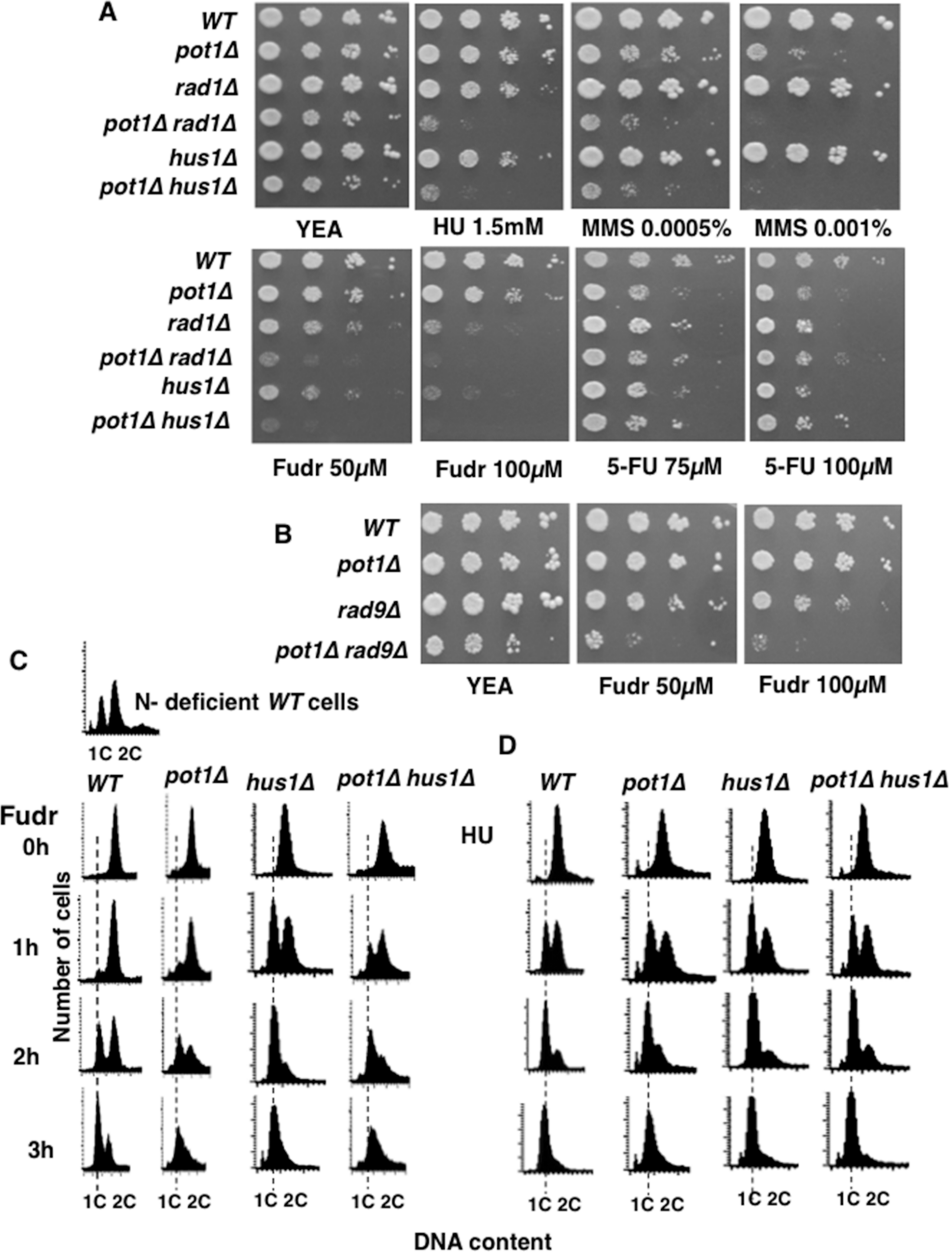
*hus1Δ and rad1Δ* cells are sensitive to HU, MMS, and Fudr especially in the absence of Pot1. **(A)** Drug sensitivity of wild-type (WT), *pot1****Δ***, *rad1****Δ***, *pot1****Δ*** *rad1****Δ***, *hus1****Δ***, and *pot1****Δ*** *hus1****Δ*** cells was determined using a spot assay. Logarithmically growing *S*. *pombe* were serially diluted 10-fold and spotted onto YEA plates as the control and on YEA plates containing HU, MMS, Fudr, or 5-FU at the indicated concentrations. The plates were incubated at 30°C for four days. **(B)** Sensitivity of *pot1****Δ*** *rad9****Δ*** double mutants to Fudr. WT *pot1****Δ***, *rad9****Δ***, and *pot1****Δ*** *rad9****Δ*** cells were assayed as on YEA plates containing Fudr. **(C-D)** FACS analysis of cell cycle progression of WT, *pot1****Δ***, *hus1****Δ***, and *pot1****Δ*** *hus1****Δ*** double mutant cells incubated with 300 μM Fudr and 12 mM HU for 1, 2 and 3 h at 30°C. The data of WT cells arrested in G1 phase by nitrogen starvation are shown above the WT data.

We assayed the sensitivity of strains with circular chromosomes (*pot1****Δ***), checkpoint defective (*hus1****Δ*** and *rad1****Δ***) and strains with circular chromosomes that were also checkpoint defective (*pot1****Δ*** *hus1****Δ*** and *pot1****Δ*** *rad1****Δ***) to differently types of replication stress, namely hydroxyurea (HU), methyl-methanesulfonate (MMS), Fudr and 5-Fluorouracil (5-FU) treatment by plating serial dilutions of the cells on the selective plates (Fig 2A). HU inhibits the synthesis of class I ribonucleotide reductase, which is responsible for the synthesis of dNTPs. Depletion of dNTP pools via HU treatment causes replication fork arrest and subsequent genomic instability [24]. Similarly, MMS modifies both guanine to 7-methylguanine and adenine to 3-methyladenine, which leads to replication blocks [25,26]. *pot1****Δ*** cells were more sensitive to MMS than the checkpoint defective strains with linear chromosomes, showing strains with circular chromosomes to be very sensitive to damage in S phase. The checkpoint-defective circular strains *pot1****Δ*** *hus1****Δ*** and *pot1****Δ*** *rad1****Δ*** double mutant cells exhibited greater sensitivity than that shown by the single mutants *pot1****Δ***, *hus1****Δ***, and *rad1****Δ***. This was also the case after HU treatment and, unexpectedly, Fudr treatment. To confirm this we analyzed the effect of loss of the third component of the 9-1-1 checkpoint complex, Rad9. Checkpoint-defective *pot1****Δ*** *rad9****Δ*** double mutant cells with circular chromosomes were similarly more sensitive to Fudr than the single mutants (Fig 2B). Together these data imply that Fudr causes DNA replication stress, even though fission yeast does not express thymidine kinase. In contrast, *pot1****Δ*** *hus1****Δ*** and *pot1****Δ*** *rad1****Δ*** double mutant cells were not more sensitive to 5-FU compared to each single mutant (Fig 2A).

The result that *pot1****Δ*** *hus1****Δ*** and *pot1****Δ*** *rad1****Δ*** double mutants were very sensitive to Fudr was intriguing. Fudr is converted to FdUMP by thymidine kinase (TK) but yeast such as *S*. *pombe* has no thymidine kinase (*tk* gene). To further investigate the mechanism of Fudr we performed FACS analysis of cell cycle progression in wild-type, *pot1****Δ***, *hus1****Δ***, and *pot1****Δ*** *hus1****Δ*** cells prior to (0 h) and following (3 h) treatment with 300 μM Fudr. In fission yeast FACS analysis is complicated. Cells spend about 60% of the cell cycle in G2, G1 is very brief and S phase is coincident with septation so the two daughter cells appear as a single unit. Thus, an asynchronous culture appears as a mainly 2C peak and cells progress from G2 (2C), mitosis (2C), through G1 (2x1C) and in S phase transiently becomes 4C (2x2C) before returning to 2C following separation of the cells in early G2. Cells from all strains (wild-type, *pot1****Δ***, *hus1****Δ***, and *pot1****Δ*** *hus1****Δ***) arrested in early S phase after 3 h in Fudr (Fig 2C), similarly to cells treated with HU (Fig 2D). Therefore, we concluded from these data that Fudr induces DNA replication arrest in *S*. *pombe* cells.

### *pot1Δ hus1Δ* cells exhibit a high frequency of chromosome segregation defects in the presence of Fudr or HU

**Fig 3:**
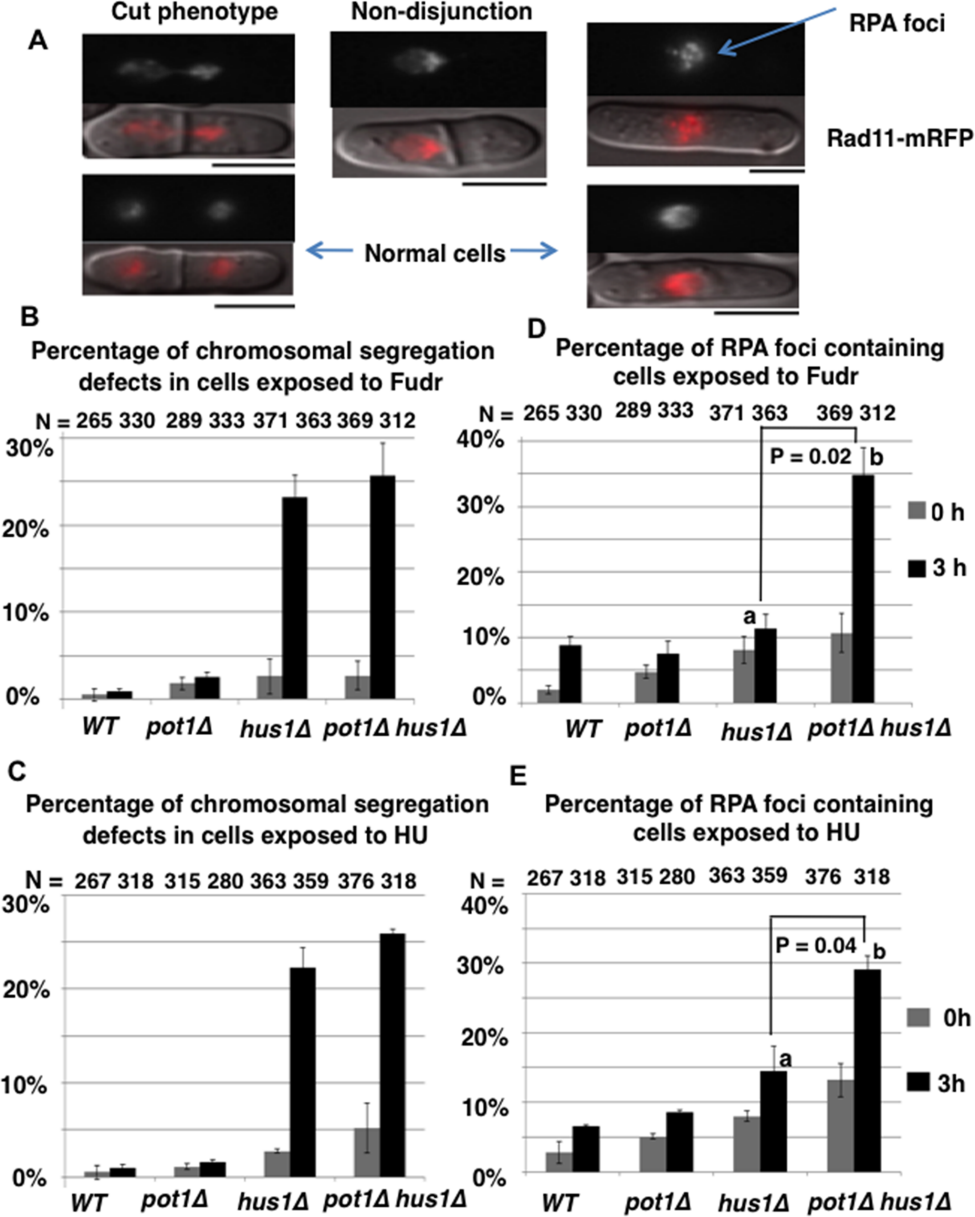
Fudr treatment induces chromosome segregation defects and RPA foci. **(A)** Representative images of chromosome segregation defects (left and middle panels), RPA foci (upper right panel) and normal fluorescence micrograph (lower right panel) of Rad11-mRFP expressing *hus1****Δ*** cells after 3 h incubation with Fudr are shown. Top image RFP, bottom image, RFP and DIC merged image. Examples of cut phenotype, where the septum bisects the nucleus (upper left), and non-disjunction, where the chromosome fail to separate (middle panel), are shown. The bar under the image represents 5 μm. **(B-C)** Analysis of chromosome segregation defects after Fudr and HU treatment. Wild-type (WT), *pot1****Δ***, *hus1****Δ*** and *pot1****Δ*** *hus1****Δ*** strains that contain Rad11(RPA)-mRFP were incubated with 300 μM Fudr or 12 mM HU for 3 h at 30°C. All types of segregation defects were scored together. Percentages of defects in chromosome segregation in cells at time 0 and 3 h following the exposure to Fudr and HU are shown. Segregation defects were scored in two independent experiments, and the bar charts show the average values ± standard error. The *y* axis denotes the percentage of cells that showed chromosome segregation defects among the total number of cells. The numbers of cells examined (N) are shown at the top. **(D-E)** Analysis of RPA foci formation after exposure to Fudr or HU. WT, *pot1****Δ***, *hus1****Δ*** and *pot1****Δ*** *hus1****Δ*** strains that contain Rad11-mRFP were incubated with 300 μM Fudr or 12 mM HU for 3 h at 30°C. % of cells with RPA foci were scored at (0 h) and after incubating (3 h) at 30°C with 300 ***μ***M Fudr (**D**) or 12 mM HU (**E**) in two independent experiments. The bar charts show the average values ± standard error. Values a and b linked by lines are significantly different at p< 0.05 (Statistical analysis ANOVA single factor followed by Duncan’s multiple ranges for multiple comparison tests). The numbers of cells examined (N) are shown at the top.

Next we investigated the effect of Fudr on chromosome segregation using Rad11 (large subunit of RPA)-mRFP expressing cells. RPA binds single strand DNA. Rad11-GFP localizes to foci corresponding to single strand regions of DNA and in addition unbound protein is nuclear allowing us to monitor chromosome segregation under the same conditions. After 3 h incubation with 300 μM Fudr, *hus1****Δ*** and *pot1****Δ*** *hus1****Δ*** cells had increased chromosome segregation defects of 8.9 fold and 9.5 fold, respectively, compare to *pot1****Δ*** cells and 23.2 fold and 25.7 fold, respectively, compare to wild-type cells (Fig 3B). Similar chromosome segregation defects were seen after exposure to 12 mM HU for 3 h (Fig 3C). These results suggest that Fudr, like HU, causes replication stress and that *hus1*^*+*^, which, as part of the 9-1-1 complex, is required for both the DNA damage and intra S phase checkpoints, is essential for the proper segregation of both linear and circular chromosomes after replication stress.

### *pot1Δ hus1Δ* cells exhibit increased levels of ssDNA in the presence of Fudr or HU

To further investigate the effect of Fudr we monitored levels of ssDNA. Rad11 (large subunit of RPA) accumulates at the site of DNA damage or replication arrest [17,27]. In untreated conditions the numbers of cells with RPA foci was increased in *pot1****Δ***, *hus1****Δ*** single and *pot1****Δ*** *hus1****Δ*** double mutants, indicating increased levels of DNA damage or replication stress. The percentage of cells containing RPA foci after 3 h incubation with 300 μM Fudr or 12 mM HU increased in all backgrounds but most significantly in the *pot1****Δ*** *hus1****Δ*** double mutant, which showed a 3 fold and 2 fold increase, respectively, compared to *hus1****Δ*** single mutant (Fig 3D and 3E). The increase in cells with RPA foci likely represents an increase in DNA lesions containing ssDNA after stalling of DNA replication forks [28]. We conclude that both Fudr and HU cause S-phase associated DNA damage in checkpoint defective cells and this is increased in the absence of *pot1*^*+*^ when chromosomes are circular.

### S phase progression in the presence of Fudr causes chromosome segregation defects in the absence of the intra S phase checkpoint

**Fig 4:**
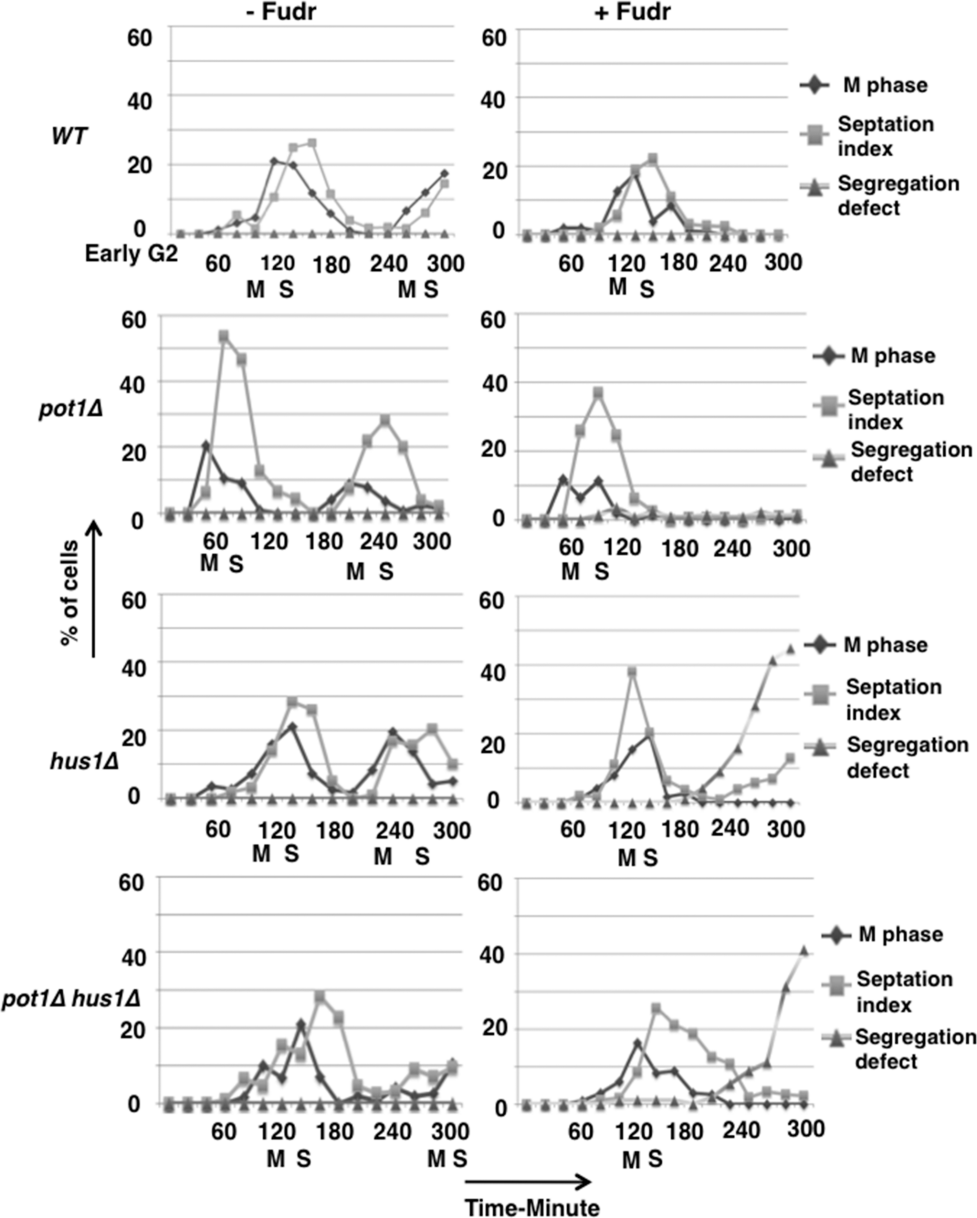
Fudr causes chromosome segregation defects only after S phase progression in *hus1Δ* and *pot1Δ hus1Δ* cells. Wild-type (WT), *pot1****Δ***, *hus1****Δ***, and *pot1****Δ*** *hus1****Δ*** cells were synchronized using lactose gradient and early G2 cells were incubated YEA liquid medium with Fudr 300 μM at 30°C, and cell cycle progression analyzed using DAPI staining and septation index in every 20 min from 0 to 300 min. The *y* axis denotes the percentage of cells that showed M phase cells, septation index and chromosome segregation defects among the total number of cells.

Next, we asked whether S phase progression is necessary for the chromosome segregation defect in *hus1****Δ*** *and pot1****Δ*** *hus1****Δ*** cells in the presence of Fudr. We synchronized cells using lactose gradient to obtain early G2 phase cells, incubated in YEA liquid medium with Fudr 300 μM at 30°C, and analyzed cells in every 20 min from 0 to 300 min. Using diamidino 2- phenylindole (DAPI) staining, we determined that in *hus1****Δ*** and *pot1****Δ*** *hus1****Δ*** cells the percentage of chromosome segregation defects did not increase in the first M phase after release (i.e. before S phase) but increased in second M phase (i.e. after S phase) compared to wild-type and *pot1****Δ*** cells (Fig 4). Wild-type, *pot1****Δ***, *hus1****Δ*** and *pot1****Δ*** *hus1****Δ*** cells did not show chromosome segregation defect in the absence of Fudr. These results suggest that Fudr exposure causes problems in S phase in *hus1****Δ*** and *pot1****Δ*** *hus1****Δ*** cells, lacking the intra S phase checkpoint, which induces chromosome segregation defects in the subsequent M phase.

### ssDNA is induced in S phase in the presence of Fudr in *pot1Δ hus1Δ* cells

**Fig 5:**
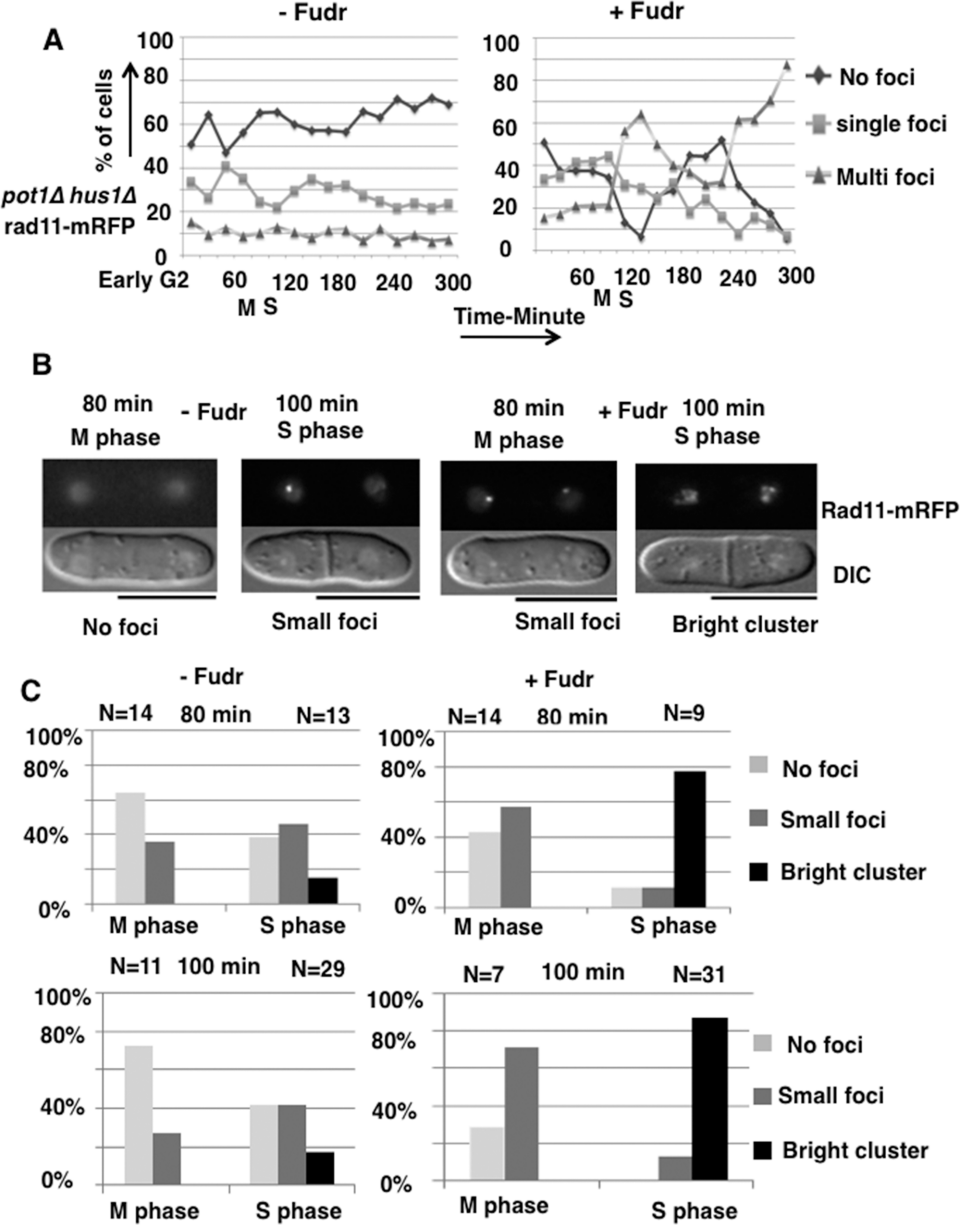
Fudr induces ssDNA in S phase in *pot1Δ hus1Δ* cells. **(A)** The *pot1Δ hus1Δ* cells that contained Rad11-mRFP were analyzed for RPA foci. Cells synchronized in early G2 using lactose gradients were incubated in YEA liquid medium with Fudr 300 μM at 30°C and samples were taken for RPA foci analysis every 20 min from 0 to 300 min. The *y* axis indicates the percentage of cells that exhibited no foci, single foci and multi foci among the total number of cells. **(B)** Representative mitotic (M phase) (binucleates without septum) and S phase (binucleates with septum) cells with no RPA foci, small RPA foci and bright RPA cluster foci in the absence and presence of Fudr at time points 80 min and 100 min after release from early G2 phase. The bar under the image represents 10 μm. **(C)** Number of no foci, small foci and bright cluster foci counted in M phase and S phase cells at 80 min and 100 min after release from early G2.

Lastly, we addressed when ssDNA increased in *pot1****Δ*** *hus1****Δ*** cells in the presence of Fudr. Cells were synchronized in early G2 phase and incubated in YEA liquid medium with Fudr 300 μM at 30°C, and analyzed cells in every 20 min from 0 to 300 min. Fudr exposure increased the percentage of cells containing clusters of bright RPA foci at time point 100 min (M or S phase) in *pot1Δ hus1Δ* cells compared to untreated cells (Fig 5A). To understand exactly when RPA foci increase, we analyzed the percentages of cells with clusters of bright foci in M phase (binucleate, no septum) and S phase (septated binucleate) cells separately at 80 min and 100 min. Clusters of bright foci were observed only in S phase, and Fudr exposure increased the numbers of cells with clusters of bright foci about 4 fold compared to untreated cells (Fig 5C). We therefore concluded that Fudr induces DNA damage in S phase in *pot1Δ hus1Δ* cells.

## Discussion

Circular chromosomes in eukaryotic cells are unstable and this instability induces chromosome lost or rearrangement, which can result in other genomic imbalances and detrimental phenotypes. Examples include ring chromosome 20 in humans that is associated with epilepsy which causes abnormal electrical activity in the brain [29]. Also, the cells from atypical lipomatous tumors contain about 85% circular chromosomes and in dermatofibrosarcoma protuberans tumor cells contain approximately 70% circular chromosomes [30,31]. In these cases, therapies targeting the cells with circular chromosomes may facilitate the selective killing. However, factors affecting the stability of circular chromosomes have not been well studied. Therefore, we generated checkpoint defective strains with circular chromosomes (*pot1Δ hus1Δ* and *pot1Δ rad1Δ* double mutant) and found that these strains were more sensitive to HU and MMS than either strains with circular chromosomes (*pot1Δ*) or checkpoint defective single mutant (*hus1Δ*). This finding revealed that the DNA damage checkpoint plays important roles in the maintenance of circular chromosomes when DNA replication has been compromised. Notably, we found that Fudr also killed the *pot1Δ* and 9-1-1 complex double mutant cells with a high frequency compared to single mutant cells. The 9-1-1 complex consists of *rad9*, *rad1* and *hus1* and we found *pot1Δ rad9Δ* double mutants also exhibited synthetic lethality in the presence of Fudr (Fig 2B), demonstrating that the 9-1-1 complex is required to maintain of circular chromosomes when DNA replication has been compromised.

The effects of Fudr in *S*. *pombe* are not well understood. As *S*. *pombe* does not express thymidine kinase, Fudr may not be converted to FdUMP efficiently. Accordingly, wild-type *S*. *pombe* cells show only a slight decrease in growth rate on Fudr, however they cannot grow when thymidine kinase is expressed. Thus, Fudr has been used for counter selection to select strains that have lost the plasmid expressing thymidine kinase [32]. Furthermore, it has been reported that Fudr inhibits DNA synthesis and increases the recombination frequency in *S*. *pombe* without ectopic expression of thymidine kinase [33,34]. These findings are consisting with our result showing that Fudr affects *S*. *pombe* without thymidine kinase expression.

5-FU and Fudr are both utilized as anticancer drugs. 5-FU is used for colon and breast cancer, and Fudr for colon and ovarian cancer [2,4]. 5-FU is converted intracellularly to the active metabolites FdUMP and fluorouridine triphosphate (FUTP) [35]. FdUMP disrupts the action of TS and hampers DNA metabolism. Fudr follows the same mechanism as FdUMP and impedes the synthesis of DNA [36,37]. Alternatively, FUTP directly disrupts RNA synthesis by inhibiting the processing of pre-rRNA into mature RNA, [38,39] post-transcriptional modification of tRNAs, [40,41] and the assembly and activity of snRNA/protein complexes, thereby inhibiting splicing of pre-mRNA [42,43]. In this study, we found that the *pot1Δ* and 9-1-1 complex double mutant cells were shown to exhibit severe sensitivity to Fudr but not 5-FU. This might be due to a dependence on the target site of *S*. *pombe* cell toxicity and suggests that Fudr affects DNA replication and repair but not RNA metabolism. Fudr induced S phase arrest in all strains tested in this work (Fig 2C), further suggesting that Fudr exposure results in incomplete DNA replication. Incomplete DNA replication also causes chromosome segregation problems because of the physical link between sister chromatids and consistent with this, exposure to Fudr resulted in chromosome segregation defects in both *hus1Δ* and *pot1Δ hus1Δ* cells. Fudr exposure induced multiple RPA foci in *pot1Δ hus1Δ* double mutant cells compare to *hus1Δ* single mutant cells (Fig 3D) and clusters of bright RPA foci were observed in S phase not M phase (Fig 5C). These results suggest that Fudr induces DNA lesions containing ssDNA in *pot1Δ hus1Δ* double mutant cells during S phase [28] and leads to increased lethality in checkpoint-defective cells with circular chromosomes compared to checkpoint-proficient cells with circular chromosomes and checkpoint-defective cells with linear chromosomes.
